# Ovarian cancer in younger *vs* older women: a population-based analysis

**DOI:** 10.1038/sj.bjc.6603601

**Published:** 2007-02-06

**Authors:** J K Chan, R Urban, M K Cheung, K Osann, A Husain, N N Teng, D S Kapp, J S Berek, G S Leiserowitz, J Y Shin

**Correction to**: *British Journal of Cancer* (2006) **95**, 1314–1320. doi: 10.1038/6603457

Owing to an error on the author's part, the last named author (JY Shin) was omitted from the author listing when the paper was originally published. The full, correct author listing is now shown above.

