# Ovarian cancer in younger *vs* older women: a population-based analysis

**DOI:** 10.1038/sj.bjc.6603757

**Published:** 2007-05-01

**Authors:** J K Chan, R Urban, M K Cheung, K Osann, J Y Shin, A Husain, N N Teng, D S Kapp, J S Berek, G S Leiserowitz

**Correction to:** *British Journal of Cancer* (2006) **95**, 1314–1320. doi: 10.1038/6603457 (published Corrigendum, *British Journal of Cancer* (2007) **96**, 534. doi: 10.1038/sj.bjc.6603601).

Owing to a publishing error, the revised author listing for the above paper was incorrectly ordered. JY Shin should have been added as the fifth, and not final, author – we are happy to correct this mistake. The complete and correct author listing is shown above.

